# An Aggregate Signature Scheme Based on a Trapdoor Hash Function for the Internet of Things

**DOI:** 10.3390/s19194239

**Published:** 2019-09-29

**Authors:** Hong Shu, Fulong Chen, Dong Xie, Liping Sun, Ping Qi, Yongqing Huang

**Affiliations:** 1 School of Computer and Information, Anhui Normal University, Wuhu 241002, China; shuhongtl@126.com (H.S.); xiedong@ahnu.edu.cn (D.X.); slp620@163.com (L.S.); 2 Anhui Provincial Key Lab of Network and Information Security, Wuhu 241002, China; 3 School of Mathematics and Computer, Tongling University, Tongling 244061, China; qiping929@gmail.com (P.Q.); hyq@tlu.edu.cn (Y.H.); 4 Institute of Information Technology & Engineering Management, Tongling University, Tongling 244061, China

**Keywords:** Internet of Things (IoT), aggregate signature, trapdoor hash function, elliptic curve discrete logarithm, random oracle model

## Abstract

With the rapid development of the Internet of Things (IoT), it becomes challenging to ensure its security. Identity authentication and integrity verification can be achieved by secure hash functions and digital signature algorithms for IoT applications. In order to solve the issues of bandwidth limitation and computational efficiency of secure communication in IoT applications, an aggregate signature scheme based on multi- trapdoor hash function is proposed in this paper. Firstly, to prevent key exposition, based on the elliptic curve discrete logarithm problem (ECDLP), we constructed a double trapdoor hash function (DTH) and proved its reliability. Secondly, the multi-trapdoor hash function (MTH) based on DTH is presented. Finally, an MTH-based aggregate signature scheme (MTH-AS) with constant signature length is proposed. Based on the assumption of ECDLP, the proposed scheme is proven unforgeable against adaptive chosen message attacks with the Forking Lemma. Different from the most signature schemes with bilinear mapping, the proposed scheme has higher computational efficiency and shorter aggregate signature length. Moreover, it is independent of the number of signers. Security analysis and performance evaluation has revealed that the proposed scheme is an ideal solution for secure IoT applications with limited computing power, storage capacity, or limited bandwidth, such as wireless sensor networks, vehicular ad hoc networks, or healthcare sensor networks.

## 1. Introduction

With the development of wireless communication technology, sensor network, microchip technology, and pervasive computing, the Internet of Things (IoT) has been applied in more and more areas, including smart home, smart health, wearable equipment, vehicular ad-hoc network, environmental monitoring, and smart grids, etc. [1]. The IoT collects various information from the physical world through radio frequency identification (RFID) devices, infrared sensors, GPS, sensors, etc. It enables people-to-people, people-to-thing, thing-to-thing connection, and communication, which in turn realizes intelligent perception, recognition, decision-making, and control on the physical world [2]. For example, wearable systems can effectively provide patients with seamless monitoring, remote surgery, telemedicine, timely assistance, and turn hospital-centered medical services into patient-centered care [3]. However, while the Internet of Things provides people with more and more convenient services, its security issues are becoming increasingly prominent. Data security and privacy preservation are major challenges in the applications of the IoT. Integrity, non-repudiation and authenticity have become the key security requirements for the Internet of Things [4].

In IoT applications, identity authentication, encryption, and integrity verification can be achieved by secure hash functions and digital signature algorithms which can ensure data privacy and rooting security [2]. Many scholars have been working on this issue [4,5,6]. Yeh et al. [5] proposed a certificateless signature scheme based on elliptic curve cryptography (ECC). Since ECC is more efficient than bilinear mapping in terms of computational efficiency [7], the scheme provides a safe and efficient interaction for IoT-based smart objects. When tackling the computational efficiency problem, Kumar et al. [6] did not select bilinear pairing operations either. Instead, they proposed a lightweight digital signature scheme based on quadratic residual theory and proved the security of the scheme under the standard model. The solution, based on the intrusion detection system (IDS), authenticates the crowdsensing data acquired from IoT environment. Meanwhile, to meet security requirements of data integrity and authenticity in IoT environment, Yang et al. [4] proposed a certificateless signature scheme. Based on the collision resistant hash function and computational Diffie-Hellman (CDH) assumption, Yang’s scheme was proved to be highly unforgeable under adaptive chosen message attacks in the standard model.

**Figure 1:**
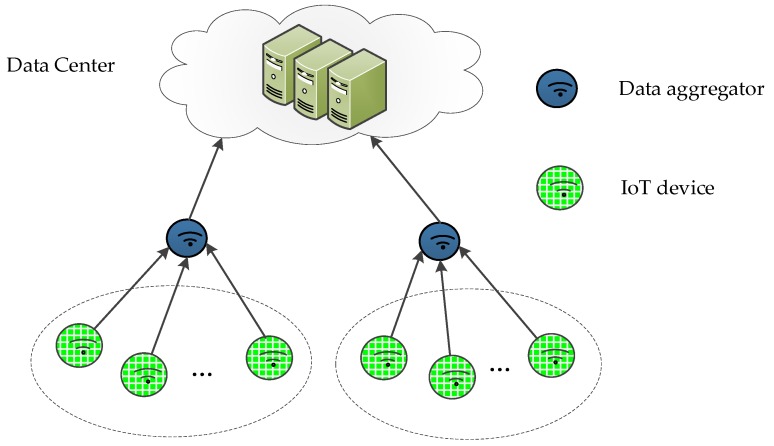
Many-to-one IoT.

Computing power, battery capacity, and storage resources are the important factors that limit IoT capabilities [2]. In IoT, such as SIoT [8,9,10] and WIoT [3], hundreds and thousands smart objects are connected. A large number of applications are many-to-one. That is, multiple data senders and one data receiver. Figure 1 shows the many-to-one IoT scenario. In the scenario, smart objects generate or collect information from the physical world and pass data from one node to the other. Finally, the receiver aggregates IoT data and sends it to data center. In the case of smart health, a patient can generate multiple medical records. Some medical records, blood pressure, blood sugar, heart rate, etc., are generated from wearable devices. Other medical records such as medical orders and prescriptions, are from medical personnel. Considering the integrity and authenticity of medical data, each individual medical record should be signed by the corresponding responsible entity. Consequently the digital signatures on different medical data will map to one patient (many-to-one). Aggregate signature can compress the digital signatures of different messages into one short digital signature, thus saving storage space and improving computational efficiency. It is very suitable for IoT applications such as vehicular ad-hoc network, smart grid and wireless sensor network with limited bandwidth and computational resources. This nature of aggregate signatures has attracted interests of more and more scholars [11,12,13]. Pankaj Kumar et al. [11] proposed a certificateless aggregate signature scheme for medical wireless sensor networks. This scheme features certificateless cryptosystem and aggregate signature, and preserves privacy, non-repudiation and integrity of medical wireless sensor networks. In regard to vehicle-to-infrastructure (V2I) communication in vehicular ad hoc networks, Horng et al. [12] proposed a certificateless aggregate signature. It implements conditional privacy preservation through pseudo identity. Another aggregate signature scheme was proposed by Shen et al. [13] for wireless sensor networks based on identity-based cryptography. That solution has been proved resistant to coalition attacks, capable to reduce energy consumption and ensure security for data acquisition, processing and transmission in wireless sensor networks.

The motivation of our study is to find a solution to identity authentication and integrity verification in many-to-one IoT scenarios where device resources are limited, such as smart health, vehicular ad hoc networks and smart grid et al. In these applications, traditional encryption, authentication and cryptographic algorithms can severely reduce the efficiency of small embedded devices and increase their power consumption. However, secure hash function and lightweight aggregate signature algorithm can effectively solve the problem of battery capacity, computing power and storage capacity limitation while fulfilling security requirements. Therefore, we propose an aggregate signature scheme based on multi-trapdoor hash function in this paper.

The contributions of this paper are as follows:

- Based on ECDLP, we constructed a double trapdoor hash function and a multi-trapdoor hash function respectively. Batch trapdoor collision computation of multi-trapdoor hash function can improve the efficiency of aggregate signature.
- An aggregate signature scheme based on MTH is proposed. With Forking Lemma, the proposed scheme is proven to be secure against the existing unforgeability on adaptively chosen message attacks.
- Compared with other bilinear pairings-based schemes, our ECC-based scheme is more efficient in terms of computational overhead. On the other hand, our MTH-AS scheme has the advantage in storage capacity because the length of the proposed aggregate signature is a constant.
- Due to the above performance, our MTH-AS scheme is suitable for secure IoT applications with limited computing power, storage capacity, and bandwidth.

The rest of this paper is organized as follows. Section 2 discusses the relevant works. The necessary preliminaries and system model are given in Section 3. Section 4 presents the ECDLP-based double trapdoor hash scheme DTH. Section 5 describes the ECDLP-based multi-trapdoor hash function MTH. Thereafter, we demonstrate the MTH- based aggregate signature scheme for IoTs and carry out performance comparison in Section 6. Finally, the conclusion is offered in Section 7.

## 2. Related Work

Hash functions are one-way and collision resistant. Being a special type of hash function, trapdoor hash function [14] is related to the concept of trapdoor commitment [15]. The trapdoor hash function uses some special information (the trapdoor information) to produce a fixed hash value. People who know the trapdoor information open the trapdoor commitment in different ways, thus opening different collisions [15]. That means, the owner of the trapdoor can calculate the trapdoor collision. 

Krawczyk et al. [14] first proposed the trapdoor hash function in order to construct chameleon signatures. Thereof, many digital signature schemes were developed based on chameleon signatures. One of the most representative schemes was a solution proposed by Shamir et al. [16] for online/offline signatures. The scheme could resist the adaptive chosen message attacks. However, it encountered key exposure problems of the chameleon hash. That is because collision calculation would lead to exposure of trapdoor information. Focusing on solving this problem, Chen et al. [17] and Atteniese et al. [18] proposed trapdoor hash schemes without key exposure. In 2008, Chen et al. [19] introduced a special double trapdoor hash scheme. This scheme features two trapdoors, long-term trapdoor and temporary trapdoor. It guarantees the safety of long-term trapdoor at the cost of temporary trapdoor leakage. 

Chandrasekhar and Singhal et al. [20,21,22,23] carried out in-depth study on trapdoor hash function. Based on discrete logarithm, Chandrasekhar et al. [20] proposed a multi-trapdoor hash function without key exposure. This scheme had multiple trapdoors corresponding to different entities. It was suitable for constructing multi-party digital signatures. Chandrasekhar et al. [22] put forward the concept of aggregate signcryption. They proposed a new efficient scheme of aggregate signcryption which could generate the aggregate signcryption text of constant order. The new scheme combined multi-trapdoor hash function with decomposable multiplicative homomorphism ElGamal encryption while providing confidentiality, integrity and identity authentication for many-to-one communication scenarios.

The conception of aggregate signature was first proposed by Boneh et al. [24] in 2003, which has played a significant role in promoting digital signature cryptography technology. Based on trapdoor permutations, sequential aggregate signature was proposed by Lysyanskaya et al. [25] in 2004. In this scheme, before adding his own signature, every signer has to verify all the previously aggregated signatures. It is, however, not suitable for the situation where the signers operate independently of each other. Accordingly, Brogle et al. [26] improved it with the idea of "lazy verification". Their scheme does not require the signer to know the public key of other signers, but the length of the signature grows linearly when the number of signers increases. In order to reduce the interaction between signers, a synchronous aggregation signature scheme with a synchronous clock was proposed by Ahn et al. [27]. The scheme allows a signer to sign at most once in each period of time, and only the signatures in the same period can be aggregate. However, the computation cost is relatively high. 

The identity-based aggregation signature scheme proposed by Gentry et al. [28] does not require to store the public key of each signer. The purpose is to minimize the total amount of information for verification. However, it requires an additional trusted third party (e.g., key generation center). 

Certificateless aggregation signature has the characteristics of keyless escrow in certificateless cryptosystem and relatively low computation and communication overhead in aggregation signatures. That’s why some relevant scholars have made in-depth research on it [29,30]. In 2007, Gong et al. [29] firstly proposed two certificateless identity-based aggregation signature schemes. But there were shortcomings in respect of signature length and verification efficiency. Zhang et al. [30] introduced a certain improvement in computation efficiency. In Zhang’s scheme, the verification process, not reliant on the number of aggregate signatures, requires a small set of a constant number of pairing computations. However, the generation of aggregate signatures requires assistance of a synchronous clock. 

With the Forking Lemma [31], the scheme proposed by Chen et al. [32] was proven strong security based on the hardness of computational Diffie-Hellman problem (CDHP). It makes use of the bilinear pair and state information. However, the length of signature grew with the number of signers. The scheme proposed by Li et al. [33] drew on the state information of Chen et al. [32]. It was existentially unforgeable against adaptively chosen message attacks without the Forking Lemma. The scheme provides fixed-length aggregate signatures. Zhou et al. [34] and Cheng et al. [35] effectively compensated for the shortcomings of the above two schemes [32,33] by using elliptic curve discrete logarithm problem (ECLDP). Zhou et al. [34] proposed two certificateless aggregate signatures CLAS-1 and CLAS-2, which were proven unforgeable by the discrete logarithm problem (DLP). Compared with CLAS-1, information sharing was used in CLAS-2 to aggregate partial signatures in advance. CLAS-2 provides shorter constant-level signature lengths than CLAS-1. Cui et al. [36] applied the certificateless aggregation signature scheme to vehicle ad hoc network, and used pseudo-identity to provide privacy preservation for vehicle information. The scheme has high computation efficiency. However, the length of signature is related to the number of signers.

Among the above-mentioned schemes, the aggregate signature lengths of the schemes [26,32,35,36], CAS-1 [29], and CLAS-II [34] increase linearly with the number of signers and they are only suitable for low bandwidth network environments. Meanwhile, the schemes [24,29,32] based on bilinear pairs having no advantage in the computational performance because the time overhead of bilinear pair operations is relatively high [7]. The comparisons of relevant aggregate signatures are shown in Table 1.

## 3. Preliminaries

### 3.1. Symbolic Representation

The symbols used in the proposed scheme are shown in Table 2.

### 3.2. Double Trapdoor Hash Function

Different from other hash functions, trapdoor hash function is a probability function with the hash key and the trapdoor key < *HK*, *TK* >. The collision resistance of the trapdoor hash functions depends on the user’s knowledge state [16]. When both *TK* and *HK* are known, it is easy to calculate the trapdoor collision. That is to say, when only *HK* is known, it is difficult to find two different messages *M* and *M*′ in the message space, and two different auxiliary parameters *R* and *R*′, which satisfy ${TH}_{HK}\left(M,\;R\right){=TH}_{HK\;}\left(M{}^{\prime },\;R^{\prime }\right)$. However, when both *TK* and *HK* are known, it is easy to calculate *R*′ based on *M*, *M*′ and *R* such that ${TH}_{HK}\left(M,\;R\right){=TH}_{HK\;}\left(M{}^{\prime },\;R{}^{\prime }\right)$.

In trapdoor hash function, the calculation of the collision causes the trapdoor key to leak, which is called key exposure. In chameleon signature, the exposure of the trapdoor key affects its transitivity. In online/offline signature scheme, key exposure will result in anyone being able to forge the signature. A hash function without key exposure usually has two trapdoors, i.e., a long-term trapdoor and a temporary trapdoor. Collision calculations only expose the temporary trapdoor, thus preventing the long-term trapdoor key from leaking.

Different from the traditional trapdoor hash function family [16], the trapdoor hash function [20] proposed in this paper is a variant of the double trapdoor hash function family [21], using the temporary key pair < *HK*′, *TK*′ > to generate trapdoor collisions.

**Definition** **1.** *Trapdoor hash function consists of four probability polynomial time (PPT) algorithms **< ParGen, KeyGen, HashGen, TrapColGen >**.*

- ***ParGen*** *: Inputs security parameter k, outputs system parameters Params;*
- ***KeyGen*** *: Inputs Params, outputs < HK, TK >;*
- ***HashGen*** *: Inputs Params, message M, and auxiliary parameter R, and outputs the trapdoor hash value ${TH}_{HK}\left(M,\;R\right)$;*
- ***TrapColGen*** *: Inputs Params, < HK, TK >, M, R, and new message M′(≠M), and outputs new auxiliary parameter R′ and the temporary hash key HK′ such that:*
  $${TH}_{HK}\left(M,\;R\right){=TH}_{HK{}^{\prime }\;}\left(M{}^{\prime },\;R{}^{\prime }\right);$$

*When HK ≠ HK′, < HK′, TK′ > and < HK, TK > are called temporary hash/trapdoor key pair and long-term hash/trapdoor key pair respectively. The properties of double trapdoor hash functions are as follows:*

*(1)*  *Validity: Given HK and (M, R), ${TH}_{HK}\left(M,\;R\right)$ is calculated in polynomial time.*
*(2)*  *Collision resistance: Given HK, there is no PPT algorithm that can find HK′ which satisfies:*
  $${TH}_{HK}\left(M,\;R\right){=TH}_{HK{}^{\prime }\;}\left(M{}^{\prime },\;R{}^{\prime }\right),\;M{}^{\prime }\neq M.$$
*(3)*  *Trapdoor collision: There is a PPT algorithm, given < HK, TK >, (M, R) and new message M′ ≠ M, output HK′ and R′ such that:*
  $${TH}_{HK}\left(M,\;R\right){=TH}_{HK{}^{\prime }\;}\left(M{}^{\prime },\;R{}^{\prime }\right).$$
*(4)*  *Key exposure freeness: Given the long-term hash key HK, temporary hash key HK′, and (M, R), (M′, R′), M′ ≠ M, there is no PPT algorithm to output long-term trapdoor key TK with non-negligible probability.*

### 3.3. Elliptic Curve Discrete Logarithm

**Definition** **2.** *(Elliptic curve discrete logarithm problem (ECDLP) [37]). E(F_l_) is an elliptic curve over the finite field F_l_. And P is a q-order generator of E(F_l_), when $Q\in {E(F}_{l})$ and Q = kP, find the integer k (0 ≤ k ≤ q−1).*

*This definition is also known as the onewayness of ECDLP. The probability that algorithm A successfully solves ECDLP is defined as:*

$${Adv}_{A}^{ECDLP}(\varphi )=Pr\left[A\left(\;q,\;P,\;Q\right)=\;k|0\;<\;k\;\leq \;q-1,\;Q\;=\;kP\right]$$

It is determined by the random selection of $k\;\in_{R\;}Z_{q}^{*}$ and A.

### 3.4. Aggregate Signature

Aggregate signatures consist of PPT algorithms: **AS = < Setup, KeyGen, Sign, Verify, Aggregate, Aggregate Verify >**. And the tuple **< Setup, KeyGen, Sign, Verify >** constructs a standard system parameter establishment, key generation, signature, verification of the short signature process, called the standard signature of aggregate signature.

- **Setup**: Inputs security parameter *k*, outputs system parameter *Params*.
- **KeyGen**: For a particular ${ID}_{i}\in U$ (*U* is a user set), inputs system parameter *Params*, then outputs the private and public key < *y*, *Y* >.
- **Sign**: For a message *M_i_* to be signed, inputs private key $y_{i}$, outputs individual signature $\sigma_{i}$.
- **Verify**: Inputs public key *Y_i_*, message *M_i_*, and individual short signature $\sigma_{i}$, if the verification algorithm is successful, it outputs *ACCEPT*, otherwise, it outputs *REJECT*.
- **Aggregate**: Inputs ${{\{ID}_{i\;}\}}_{1}^{n}\in U$, their signature messages ${{\{M}_{i}\}}_{1}^{n}$ and individual signatures ${{\{\sigma }_{i\;}\}}_{1}^{n}$, outputs aggregate signature σ.
- **Aggregate Verify**: Inputs public keys ${{\{Y}_{i}\}}_{1}^{n}$, messages ${{\{M}_{i}\}}_{1}^{n}$, and aggregate signature σ, if the aggregation validation algorithm is successful, it outputs *ACCEPT*, otherwise, it outputs *REJECT*.

### 3.5. Security Model

Assuming *k* is a security parameter, $G_{A}^{MTH\_AS}{(1}^{k})$ is a game between challenger B and adversary V. The attack model is shown below:

- Setup
  Inputs the security parameter *k*, B runs the *Setup* algorithm and returns the system parameter to V.
- Query
  V adaptively performs the following oracle query.
  – Hash queries: V makes hash oracle queries to all hash functions in the proposed scheme, and challenger B returns the corresponding value.
  – Trapdoor hash queries: V inputs *< m*, *r >* for trapdoor hash query and the oracle outputs ${TH}_{Y}\;(m,\;r)$.
  – Key queries: V inputs the message $m_{i}$ of user *i* to make key query, and the oracle returns the trapdoor key *y* of user *i* to the adversary V.
  – Signature queries: V inputs the original message/random value pair ${<\;m}_{i}\;{,\;r}_{i}\;>$, new message $m_{i}^{\prime }$ and hash key ${TK}_{i}$, the oracle outputs the signature.
- Forge
  Finally, V outputs $\sigma^{*}{=\;(\;K}^{*}{,\;C}^{*})$ as a forged aggregate signature based on new message set ${\left\{m_{i}^{\prime }{}^{*}\right\}}_{1}^{n}$.
- The adversary V wins the game if $\sigma^{*}$ is a valid signature and V does not make a key query on at least one user among *n* users.

### 3.6. System Model

**Figure 2:**
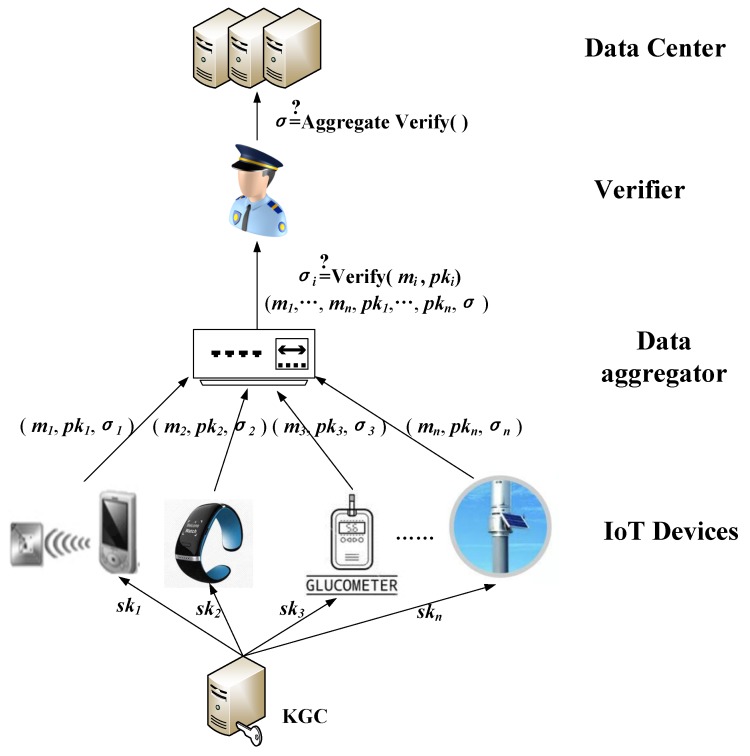
System model.

In many-to-one IoT scenarios where bandwidth, computing power, and storage resources are limited, it is important to improve computational efficiency and storage capacity. Furthermore, it is also vital to protect data from modification and repudiation. Due to its natural compression properties, aggregate signatures are ideal for resource-constrained many-to-one IoT applications. As shown in Figure 2, the system model of the aggregated signature in the IoT environment proposed in this paper consists of five components: the key generation center (KGC), IoT devices, data aggregator, verifier and data center.

• *KGC*

The KGC is responsible for system setup. It is regarded to be trusted in our proposed scheme. The KGC generates system parameters and sends them to all the entities, such as IoT devices, aggregator, verifier and data center. The private keys ${sk}_{i}$ are computed by the KGC for each IoT device. Then these private keys are sent to each entity through a secure channel.

• *IoT Devices*

The IoT devices with limited computational and storage capacity are capable to collect real data from the physical world. In order to ensure data integrity, non-repudiation, privacy, and authenticity, with the system parameter and the private key, each IoT device makes individual signature on the original data they collect. Then the IoT devices send message $m_{i}$, individual signature $\sigma_{i}$ and public key ${pk}_{i}$ to the data aggregator.

• *Data aggregator*

The data aggregator may be a node in the system model that verifies all the individual signatures it receives. It checks the validity of the individual signatures, if they are correct, then aggregates them into a single short signature. Finally, the data aggregator sends the aggregate signature to the verifier.

• *Verifier*

The verifier are responsible to check the correctness of the received aggregate signature. It can verify the correctness of all individual signatures by one operation. If the aggregation signature is verified correctly, all the messages and the aggregate signature are sent to the data center.

• *Data Center*

The data center has powerful storage space and computing power, which can store and share the validated aggregate signatures and original messages safely.

## 4. Scheme of Double Trapdoor Hash Function

### 4.1. Double Trapdoor Hash Scheme Based on ECDLP

In this section, a scheme of double trapdoor hash function based on ECDLP is presented, which is consisted of the tuple: **DTH = < DParGen, DKeyGen, DHashGen, DTrapColGen >**.

- **DParGen**: Select *l* and a big prime *p*, where ${l\;=\;p}^{m}$. Let *E*(*F_l_*) be an elliptic curve over finite field *F_l_* and *G* a cyclic subgroup of *E*(*F_l_*). Let *P* be a generator of *G* with prime order *q* and ${H\;:\;\{0,\;1\}}^{*}\;\to {\;Z}_{q}^{*}$, ${f\;:\;G\;\times \;Z}_{q}^{*}\;\times \;G\;\to {\;Z}_{q}^{*}$, $F\;:\;G\;\to {\;Z}_{q}^{*}$ cryptographic hash functions. The system parameters are *params* = < *G*, *P*, *q*, *H*, *F*, *f* >.
- **DKeyGen**: Select randomly $y\;\in {\;Z}_{q}^{*}$ and compute Y = yP. The trapdoor key is *y* and the hash key is *Y*.
- **DHashGen**: Select randomly $t\;\in {\;Z}_{q}^{*}$, compute A = tP and r = F(A). The trapdoor hash value is h = H(m)P + rY.
- **DTrapColGen**: Select randomly $t{}^{\prime }\;\in {\;Z}_{q}^{*}$, compute:
  $$A{}^{\prime }\;=\;t{}^{\prime }\;P\;\mathrm{and}\;r{}^{\prime }\;=\;F(A{}^{\prime }\;).$$
  The temporary trapdoor key is ${y{}^{\prime }\;=\;r{}^{\prime }}^{-1}\;(H(m)\;-\;H(m{}^{\prime })\;+\;ry\;)\;\mathrm{mod}\;q$ and the temporary hash key is $Y{}^{\prime }\;=\;y{}^{\prime }\;P$.
  Compute:
  $$k\;=\;t{}^{\prime }-\;y\;*\;f\;(h\;,r{}^{\prime }\;,\;Y{}^{\prime }\;)\;\mathrm{mod}\;q.$$
  which then outputs $<\;k\;,\;r{}^{\prime },\;Y{}^{\prime }\;>$.
  $<\;k\;,\;r{}^{\prime }>$ is the signature on ${TH}_{HK}\left(m,\;r\right){=TH}_{HK{}^{\prime }\;}\left(m{}^{\prime },\;r{}^{\prime }\right)$ verifiable under *Y* [20]. The verification equation expands:
  $$\begin{array}{l} \;kP\;+\;f\;(\;h,\;r{}^{\prime },\;Y{}^{\prime })\;Y\;\\ =\;(\;t{}^{\prime }-\;y\;*\;f\;(\;h,\;r{}^{\prime },\;Y{}^{\prime }))\;P\;+\;f\;(\;h,\;r{}^{\prime },\;Y{}^{\prime })\;*\;yP\\ =\;t{}^{\prime }P\\ =\;A{}^{\prime }\\ F\;(A{}^{\prime })\;=\;r{}^{\prime } \end{array}$$

### 4.2. Security Analysis

(1) *Efficiency*: Given the system parameter *params*, the hash key *Y* and the message/auxiliary parameter pair < *m*, *r* >, the trapdoor hash valve h = H( m ) P + rY is computable in PPT.
(2) *Trapdoor collisions*: Given < *y*, *Y* >, < *m*, *r* > and new message $m{}^{\prime }(\neq \;m)\;\in {\;\{0,1\}}^{*}$, choose randomly $t{}^{\prime }\;\in {\;Z}_{q}^{*}$. Then compute
  $$A{}^{\prime }\;=\;t{}^{\prime }\;P,\;r{}^{\prime }\;=\;F(A{}^{\prime }\;).$$
  The temporary trapdoor key is given by
  $${y{}^{\prime }\;=\;r{}^{\prime }}^{-1}\;(H(m)\;-\;H(m{}^{\prime })\;+\;ry\;)\;\mathrm{mod}\;q$$
  which satisfies
  $$H(m)P\;+\;rY\;=\;H(m{}^{\prime })P\;+\;r{}^{\prime }Y{}^{\prime }.$$
  That is to say
  $$H(m)\;+\;ry\;=\;H(m{}^{\prime })\;+\;r{}^{\prime }y{}^{\prime }.$$
(3) *Key exposure freeness*: Given two tuples < *Y*, *m*, *r* > and < *Y*’, *m*’, *r*’ > such that:
  $${TH}_{Y}\left(m,\;r\right){=TH}_{Y{}^{\prime }\;}\left(m{}^{\prime },\;r{}^{\prime }\right).$$
  That is to say:
  $$H(m)\;+\;ry\;=\;H(m{}^{\prime })\;+\;r{}^{\prime }y{}^{\prime }.$$
  In the equation, the long-term trapdoor key *y* is not computable because $y{}^{\prime }$ is unknown. That is, the computation complexity of $y{}^{\prime }$ is equivalent to ECDLP because $y{}^{\prime }$ is solved by $Y{}^{\prime }\;=\;y{}^{\prime }\;P$.
(4) *Collision resistance*: The PPT collision forger E is assumed to resist the DTH scheme with a non-negligible probability. Given *params* and *HK*, E runs in polynomial time and outputs $<\;m,\;r,\;m{}^{\prime },\;r{}^{\prime },\;HK{}^{\prime },\;k{}^{\prime }\;>$ with non-negligible probability where the following statements hold:
  $$\begin{array}{c} {TH}_{HK}\left(m,\;r\right){=TH}_{HK\;}\left(m{}^{\prime },\;r{}^{\prime }\right)\;,\;\\ F\;(k{}^{\prime }\;P\;+\;f\;(\;h,\;r{}^{\prime },\;Y{}^{\prime })Y\;)\;=\;r{}^{\prime }\;,\;\\ m{}^{\prime }\;\neq \;m,\;HK{}^{\prime }\;\neq \;HK\mathrm{and}\;r{}^{\prime }\;\neq \;r \end{array}$$
  Suppose E can construct a PPT algorithm *Q* for solving ECDLP. Given an instance of ECDLP < *G*, *P*, *q*, *Y* >, *Q* needs to find a value $z\;\in {\;Z}_{q}^{*}$ so that Z = zP. The hash function *f* acts as a random oracle $O_{f}$ that *Q* simulates. That means *Q* provides a random value for each new query to answer any hash query of $O_{f}$. Then *Q* gives two identical answers if the same query is asked twice.
  *Q* runs an instance of E and answers any hash query of $O_{f}$ until E produces collision forgery. When E queries ${<\;TH}_{Y}\left(m,\;r\right),\;r{}^{\prime },\;Y{}^{\prime }\;>$ to $O_{f}$, *Q* answers *x*’. With the Oracle replay attack [38], *Q* rewinds E to the point when E queries ${<\;TH}_{Y}\left(m,\;r\right),\;r{}^{\prime },\;Y{}^{\prime }\;>$ to $O_{f}$, and select randomly a new value $x{}^{\prime \prime }\;\neq \;x{}^{\prime }\;\in_{R}\;Z_{q}^{*}$ as the answer to E. *Q* continues running E until producing another collision forgery $<m_{2}{,\;r}_{2}{,\;m{}^{\prime }}_{2}{,\;r{}^{\prime }}_{2},\;Y{}^{\prime \prime },\;k{}^{\prime \prime }\;>$. Each instance of E is randomly selected. Given ${TH}_{HK}\left(m_{1}{,\;r}_{1}\right)$, ${TH}_{HK{}^{\prime }\;}\left(m_{2}{,\;r}_{2}\right)$, $m_{1}\;\neq {\;m}_{2}$, $r_{1}\;\neq {\;r}_{2}$, $m_{1}\;\neq {\;m{}^{\prime }}_{1}$, $r_{1}\;\neq {\;r{}^{\prime }}_{1}$, which satisfy the following equations:
  $$\begin{array}{c} \{\begin{array}{c} {\;TH}_{HK}\left(m_{1}{,\;r}_{1}\right){\;=\;TH}_{HK{}^{\prime }}\left({m{}^{\prime }}_{1}{,\;r{}^{\prime }}_{1}\;\right)\\ \;{TH}_{HK}\left(m_{2}{,\;r}_{2}\right){\;=\;TH}_{HK{}^{\prime }}\left({m{}^{\prime }}_{2}{,\;r{}^{\prime }}_{2}\;\right) \end{array}\\ \{\begin{array}{c} k{}^{\prime }\;=\;t\;-\;y\;*\;x{}^{\prime }\\ \;k{}^{\prime \prime }\;=\;t\;-\;y\;*\;x{}^{\prime \prime } \end{array} \end{array}$$
  According to these two equations, the following can be computed:
  $${y\;=\;(\;k{}^{\prime \prime }\;-\;k{}^{\prime })(x{}^{\prime }\;-\;x{}^{\prime \prime })}^{-1}.$$
  This is contrary to the elliptic curve discrete logarithm hypothesis.

## 5. Multi-Trapdoor Hash functions based on ECDLP

The multi-trapdoor hash function [20] contains many participants *U_1_*, …, *U_n_*, each of them having its own trapdoor/hash key pair ${{\{TK}_{i}\;{,\;HK}_{i}\;\}}_{1}^{n}$ and original message ${{\{m}_{i}\;\}}_{1}^{n}$. It generates the objective multi-trapdoor hash value *h* according to ${{\{m}_{i}{,\;r}_{i}\;\}}_{1}^{n}$ and ${{\{\;HK}_{i}\;\}}_{1}^{n}$, then the participants can produce hash collisions with *h* based on new messages ${{\{m{}^{\prime }}_{i}\;\}}_{1}^{n}$. The multi-trapdoor hash function combines multiple collisions generated by multiple participants to generate a single collision [20], thus saving storage space and bandwidth effectively.

### 5.1. Formal Definition

The multi-trapdoor hash function is composed of tuples **< MParGen, MKeyGen, MHashGen, MTrapColGen >**.

- **MParGen**: Inputs security parameter *k*, outputs system parameter *Params*.
- **MKeyGen**: Inputs system parameter *Params*, each participant $U_{i}$(i ∈ [1, n]) outputs ${<\;TK}_{i}\;{,\;HK}_{i\;}>$ (i ∈ [1, n]).
- **MHashGen**: Inputs *Params*, hash key group ${{\{\;HK}_{i}\;\}}_{1}^{n}$, message/auxiliary parameter pairs ${{\{m}_{i}{,\;r}_{i}\;\}}_{1}^{n}$, outputs multi-trapdoor hash value ${TH}_{{\left\{\;{HK}_{i}\;\right\}}_{1}^{n}}({\left\{{\;m}_{i}{,\;r}_{i}\;\right\}}_{1}^{n})$.
- **MTrapColGen**: Inputs *Params*, a trapdoor key ${TK}_{j}$, message/auxiliary parameter pairs ${{\{m}_{i}{,\;r}_{i}\;\}}_{1}^{n}$ and a new message ${\;m}_{j}\;\neq {\;m{}^{\prime }}_{j}$, outputs collision parameter ${<\;r{}^{\prime }}_{j}\;{,\;HK{}^{\prime }}_{j}>$ which satisfies the following equation:
  $${TH}_{{\left\{{\;HK}_{i\;}\right\}}_{1}^{n}}\left({\left\{m_{i}\;{,\;r}_{i}\;\right\}}_{1}^{n}\right){\;=\;TH}_{\{\begin{array}{c} {\;HK}_{i}\;\to {\;HK{}^{\prime }}_{i}{\;\}}_{1}^{n} \end{array}\backslash \{\;j\;\}}({\left\{m_{i}\;\to {\;m{}^{\prime }}_{i},\;{\;r}_{i}\;\to {\;r{}^{\prime }}_{i}\;\right\}}_{1}^{n}\backslash \{\;j\;\})$$

### 5.2. The ECDLP-Based Multi-Trapdoor Hash Function

This section presents an ECDLP-based multi-trapdoor hash function MTH. The algorithm process describes as follows:

- **MParGen**: Similar to *DParGen* in Section 4.
- **MKeyGen**: For each participant $U_{i}$, select randomly the long-time trapdoor key $y_{i}\;\in {\;Z}_{q}^{*}$ and compute long-time hash key:
  $$Y_{i}{\;=\;y}_{i}P.$$
  then outputs ${{\{y}_{i}\;{,\;Y}_{i}\;\}}_{1}^{n}$.
- **MHashGen**: For each $U_{i}$, select randomly $t_{i}\;\in {\;Z}_{q}^{*}$, compute auxiliary parameters:
  $$A_{i}{\;=\;t}_{i}\;P\;\mathrm{and}\;r_{i\;}{=\;F(\;A}_{i}\;).$$
  Trapdoor hash value is $h_{i}{\;=\;H(m}_{i}{)P\;+\;r}_{i}Y_{i}$. Finally, aggregate all the trapdoor hash values as multi-trapdoor hash value:
  $$h=\sum_{i=1}^{n}h_{i}$$
  then outputs *h*.
- **MTrapColGen**: For each $U_{i}$, select randomly ${t{}^{\prime }}_{i}\;\in {\;Z}_{q}^{*}$ and compute new auxiliary parameters:
  $${A{}^{\prime }}_{i}{\;=\;t{}^{\prime }}_{i}\;P\;\mathrm{and}\;{r{}^{\prime }}_{i\;}{=\;F(\;A{}^{\prime }}_{i}\;).$$
  According to trapdoor collision, compute temporary trapdoor/hash key:
  $$\begin{array}{c} {y{}^{\prime }}_{i}{\;=\;r{}^{\prime }}_{i}{}^{-1}{\;(H(m}_{i}{)\;-\;H(m{}^{\prime }}_{i}{\;)\;+\;r}_{i}y_{i}\;)\;\mathrm{mod}\;q\\ {Y{}^{\prime }}_{i}{\;=\;y{}^{\prime }}_{i}\;P\;. \end{array}$$

### 5.3. Security Analysis

**Theorem** **1.** *The proposed multi-trapdoor hash function scheme is collision resistant.*

**Proof.**  The PPT collision forger E is assumed to resist the MTH scheme with a non-negligible probability. Suppose E can construct a PPT algorithm *Q* for solving ECDLP. Given an instance of ECDLP < *G*, *P*, *q*, *Y* >, *Q* needs to find a value $z\;\in {\;Z}_{q}^{*}$ so that Z = zP.

*Q* runs an instance of E and answers any hash query of $O_{f}$ until E produces collision forgery. When E queries ${<\;TH}_{Y_{i}}\left(m_{i}{,\;r}_{i}\right){,\;r{}^{\prime }}_{i}{,\;Y{}^{\prime }}_{i}\;>$ to $O_{f}$, *Q* answers ${x{}^{\prime }}_{i}$. With the Oracle replay attack [38], *Q* rewinds E to the point when E queries ${<\;TH}_{Y_{i}}\left(m_{i}{,\;r}_{i}\right){,\;r{}^{\prime }}_{i}{,\;Y{}^{\prime }}_{i}\;>$ to $O_{f}$, and select randomly a new value ${x{}^{\prime \prime }}_{i}\;\neq {\;x{}^{\prime }}_{i}\;\in_{R}\;Z_{q}^{*}$ as the answer to E*. Q* continues running E until producing another collision forgery ${<m}_{i,2}{,\;r}_{i,2}{,\;m{}^{\prime }}_{i,2}{\;,\;r{}^{\prime }}_{i,2\;}{,\;Y{}^{\prime \prime }}_{i\;}{,\;k{}^{\prime \prime }}_{i}\;>$. Each instance of E is randomly selected. Given ${TH}_{Y_{i}}\left(m_{i,1}{,\;r}_{i,1}\right)$, ${TH}_{Y_{i}{}^{\prime }\;}\left(m_{i,2}\;{,\;r}_{i,2}\right)$, ${\;m}_{i,1}\;\neq {\;m}_{i,2}$, ${\;r}_{i,1}\;\neq {\;r}_{i,2}$, ${\;m}_{i\;,\;1}\;\neq {\;m{}^{\prime }}_{i,1}$, ${\;r}_{i,1}\;\neq {\;r{}^{\prime }}_{i,1}$, which satisfy the following equations.

$$\begin{array}{c} \{\begin{array}{c} {\;TH}_{Y_{i}}\left(m_{i,1}{,\;r}_{i,1}\right){\;=\;TH}_{Y_{i}{}^{\prime }}\left({m{}^{\prime }}_{i,1}{,\;r{}^{\prime }}_{i,1}\;\right)\\ \;{TH}_{Y_{i}}\left(m_{i,2}{,\;r}_{i,2}\right){\;=\;TH}_{Y_{i}{}^{\prime }}\left({m{}^{\prime }}_{i,2}{,\;r{}^{\prime }}_{i,2}\;\right) \end{array}\\ \{\begin{array}{c} {k{}^{\prime }}_{i}{\;=\;t}_{i}{\;-\;y}_{i}{\;*\;x{}^{\prime }}_{i}\\ {\;k{}^{\prime }{}^{\prime }}_{i}{\;=\;t}_{i}{\;-\;y}_{i}{\;*\;x{}^{\prime }{}^{\prime }}_{i} \end{array} \end{array}$$

According to these two equations, the following can be computed

$$y_{i}{\;=\;(\;k{}^{\prime \prime }}_{i}{\;-\;k{}^{\prime }}_{i}{\;)(x{}^{\prime }}_{i}{\;-\;x{}^{\prime \prime }}_{i}{\;)}^{-1}.$$

It is contrary to the elliptic curve discrete logarithm hypothesis. Thus, the proposed MTH scheme is collision resistant. □

## 6. Aggregate Signature Scheme Based on MTH

### 6.1. The Aggregate Signature Scheme Based on MTH

This section presents an aggregate signature scheme based on MTH, called MTH-AS. The algorithm is presented below.

- **AParGen**: Similar to *DParGen* in Section 4.
- **AKeyGen**: For each participant $U_{i}$, select randomly the long-time trapdoor key $y_{i}\;\in {\;Z}_{q}^{*}$ and compute long-time hash key:
  $$Y_{i}{\;=\;y}_{i}\;P.$$
  then outputs ${{\{y}_{i}{,\;Y}_{i}\;\}}_{1}^{n}$.
- **AHashGen**: For each $U_{i}$, select randomly $t_{i}\;\in {\;Z}_{q}^{*}$, compute auxiliary parameters:
  $$A_{i}{\;=\;t}_{i}\;P\;\mathrm{and}\;r_{i\;}{=\;F(\;A}_{i}\;).$$
  and computes the trapdoor hash value:
  $$h_{i}{\;=\;H(m}_{i}{)P\;+\;r}_{i}Y_{i}.$$
  Finally, aggregate all the trapdoor hash values as the multi-trapdoor hash value:
  $$h=\sum_{i=1}^{n}h_{i}$$
  Then outputs *h*.
- **ATrapColGen**: For each $U_{i}$, select the latest timestamp ${t{}^{\prime }}_{i}\;\in {\;Z}_{q}^{*}$ and compute new auxiliary parameters:
  $${A{}^{\prime }}_{i}{\;=\;t{}^{\prime }}_{i}\;P\;\mathrm{and}\;{r{}^{\prime }}_{i\;}{=\;F(\;A{}^{\prime }}_{i}\;).$$
  According to trapdoor collision, compute temporary trapdoor/hash key:
  $$\begin{array}{c} {y{}^{\prime }}_{i}{\;=\;r{}^{\prime }}_{i}{}^{-1}{\;(H(m}_{i}{)\;-\;H(m{}^{\prime }}_{i}{\;)\;+\;r}_{i}y_{i}\;)\;\mathrm{mod}\;q\\ {Y{}^{\prime }}_{i}{\;=\;y{}^{\prime }}_{i}\;P. \end{array}$$
  Computes:
  $$k_{i}{\;=\;t}_{i}{{}^{\prime }-\;y}_{i}{\;*\;f\;(h,\;r}_{i}{{}^{\prime },\;Y}_{i}{}^{\prime }\;)\;\mathrm{mod}\;q.$$
  and generates *U_i_*’s individual signature:
  $$\sigma_{i}{\;=\;(r}_{i}{{}^{\prime },\;k}_{i}).$$
  outputing ${\left\{{\;\sigma }_{i}{\;,\;Y}_{i}{{}^{\prime },\;A}_{i}{{}^{\prime },\;r}_{i}{{}^{\prime },\;t}_{i}{}^{\prime }\;\right\}}_{1}^{n}$.
- **Verify**: This algorithm verifies the correctness of the individual signature of $U_{i}$, computing:
  $$B_{i}{\;=\;k}_{i}{P+\;f\;(h,\;r}_{i}{{}^{\prime },\;Y}_{i}{{}^{\prime }\;)Y}_{i}.$$
  If the equation ${F(B}_{i}{\;)=\;r}_{i}{}^{\prime }$ holds, it accepts the participant’s individual signature and outputs *ACCEPT*, otherwise, outputs *REJECT*.
- **AggSign**: For each participant *U_j_* whose individual signature is accepted, computing:
  $$\begin{array}{c} {K\;=\;K\;+\;k}_{j}\;\mathrm{mod}\;q\\ {C\;=\;C\;+\;A}_{j}{}^{\prime }\;\mathrm{mod}\;q. \end{array}$$
  outputting the aggregate signature:
  σ = (K, C).
- **AggVerify**: Let *m* be the number of participants in the aggregate signature, that is, the number of individual signatures accepted, computing:
  $$B\;=\;KP\;+\;\sum_{j=1}^{m}{\;f\;(h,\;r}_{j}{{}^{\prime },\;Y}_{j}{{}^{\prime }\;)Y}_{j}$$
  If the equation F (B ) = F (C ) holds, accepts the aggregate signature and outputs *ACCEPT*, otherwise, outputs *REJECT*.

### 6.2. The Correctness of Aggregate Verify

The aggregate verify equation expands as follows:

$$\begin{array}{l} \;B\;=\;KP\;+\;\sum_{j=1}^{m}{\;f\;(h,\;r}_{j}{{}^{\prime },\;Y}_{j}{{}^{\prime }\;)Y}_{j}\\ \;\;=\;\sum_{j=1}^{m}{(t}_{j}{{}^{\prime }-\;y}_{j}{\;f\;(h,\;r}_{j}{{}^{\prime },\;Y}_{j}{}^{\prime }\;))P\;+\;\sum_{j=1}^{m}{\;f\;(h,\;r}_{j}{{}^{\prime },\;Y}_{j}{{}^{\prime }\;)\;y}_{j}P\\ \;\;=\;\sum_{j=1}^{m}t_{j}{}^{\prime }P\\ \;\;=\;\sum_{j=1}^{m}{\;A}_{j}{}^{\prime }\\ \;\;=\;C \end{array}$$

### 6.3. Security Proof

**Theorem** **2.** *Given an adversary makes at most $q_{f}$ f-hash queries, $q_{H}$ H-hash queries,* $q_{S}$ *signature queries,* $q_{F}$ *F-queries,* $q_{K}$ *key queries,* $q_{T}$ *trapdoor hash queries within a period t in the random oracle model, and wins the game* $G_{A}^{MTH\_AS}{(1}^{k})$ *with an non-negligible probability ε, that is, successfully forging the signature of an MTH_AS scheme. Then an algorithm B can be performed in polynomial time* $t{}^{\prime }\;\leq {\;t\;+\;O(q}_{k}{\;+\;2q}_{T}{\;+\;2q}_{s}{)T}_{ME}$ *, and solve an instance of ECLDP with probability* $\varepsilon {}^{\prime }\geq \frac{1}{10^{6}q_{k}\sqrt{{\;q}_{f}}}\varepsilon$ *. Let* $T_{ME}$ *be the run time for scalar multiplication in elliptic curve.*

**Proof.**  Given an instance of ECLDP (P, yP) ϵ G, the goal of the algorithm B is to compute *y*. Assume the hash key of $m^{*}$ is *yP*. The following is a detailed interaction process between algorithm B and adversary V.

- *Setup*: Challenger B inputs security parameters $1^{k}$, runs algorithm **AParGen**, generates system parameters *params*, and sends *params* to adversary V. B simulates hash functions random oracle $D_{H}$, $\;D_{F}$ and $D_{f}$, key random oracle $D_{K}$, trapdoor hash random oracle $D_{T}$ and signature oracle $D_{S}$ to answer all the queries from adversary V. B needs to maintain 6 lists (*L_H_*, *L_F_*, *L_f_*, *L_T_*, *L_K_*, *L_S_*), whose initial values are empty.
- *Query*: V adaptively performs the following oracle queries.
  – When V queries $D_{H}$ with $m_{i}$, B checks whether existing ${(m}_{i}{,\;H}_{i})\;\in {\;L}_{H}$ or not, if so, B sends $H_{i}$ to V. Otherwise, B selects a random $H_{i}\;\in {\;Z}_{q}^{*}$, sends $H_{i}$ to V and saves $\left(m_{i}\;{,\;H}_{i\;}\right)$ into the hash list *L_H_*.
  – When V queries $\;D_{F}$ with ${\;A}_{i}$, B checks whether existing ${(A}_{i}{,\;F}_{i}\;)\in {\;L}_{F}$ or not, if so, B returns $F_{i}$ to V. Otherwise, selects a random $F_{i}\;\in {\;Z}_{q}^{*}$, returns $F_{i}$ to V and saves ${(A}_{i}{,\;F}_{i}\;)$ into the hash list ${\;L}_{F}$.
  – When V queries $D_{f}$ with ${\;(m}_{i\;}{,\;h,\;r}_{i}{{}^{\prime },\;Y}_{i}{}^{\prime }\;)$, B checks whether existing ${\;(m}_{i\;}{,\;h,\;r}_{i}{{}^{\prime },\;Y}_{i}{{}^{\prime },\;f}_{i\;})\;\in {\;L}_{f}$ or not, if so, B returns ${\;f}_{i\;}$ to V. Otherwise, selects a random $f_{i}\;\in {\;Z}_{q}^{*}$, returns ${\;f}_{i}$ to *A* and saves ${(m}_{i\;}{,\;h,\;r}_{i}{{}^{\prime },\;Y}_{i}{{}^{\prime },\;F}_{i}\;)$ into the hash list ${\;L}_{f}$.
  – When V queries $D_{T}$ with ${(m}_{i}{,\;r}_{i})$, B checks whether existing ${(m}_{i}\;{,\;r}_{i}\;{,\;h}_{i})\in {\;L}_{T}$ or not, if so, B returns $h_{i}$ to V. Otherwise, selects a random $y_{i}\;\in {\;Z}_{q}^{*}$ and computes:
    $$h_{i}\;=\;H\left(m_{i}\;\right){P\;+\;r}_{i}y_{i}P.$$
    returning $h_{i}$ to V and saves ${(m}_{i\;}{,\;r}_{i}\;{,\;h}_{i\;})$ into the hash list $L_{T}$.
  – When V queries $D_{K}$ with $m_{i}$, B checks whether existing ${(m}_{i\;}{,\;Y}_{i}\;{,\;y}_{i})\in {\;L}_{K}$ or not, if so, B returns $y_{i}$ to V. Otherwise, if $m_{i\;}\;\neq {\;m}^{*}$, selects randomly $y_{i}\;\in {\;Z}_{q}^{*}$, $k_{i}\;\in {\;Z}_{q}^{*}$ and makes a query to $D_{f}$. If ${(m}_{i}\;{,\;*,\;*,\;*,\;f}_{i})\;\in {\;L}_{f}$, computes ${\;f}_{i}^{\;\;-1}$. Otherwise, selects randomly ${\;f}_{i}^{\;\;-1}\;\in {\;Z}_{q}^{*}$ and stores ${(m}_{i}\;{,\;*,\;*,\;*,\;f}_{i})$ into ${\;L}_{f}$, computing:
    $$y_{i}=\left(t_{i}{{}^{\prime }\;-\;k}_{i}\right){\;f}_{i}^{\;\;-1}\;\mathrm{and}\;Y_{i}{\;=\;y}_{i}\;P\;.$$
    then returns ${(y}_{i}\;{,\;Y}_{i})$ to V and saves ${(m}_{i\;}{,\;Y}_{i}\;{,\;y}_{i})$ into the hash list $L_{K}$. If $m_{i\;}{=\;m}^{*}$ holds, the game is over and outputs ∇.
  – When V queries $D_{S}$ with ${(TK}_{i}{,\;m}_{i}\;\;{,\;m}_{i}{}^{\prime }\;)$, B checks whether existing ${(\;m}_{i}\;{,\;m}_{i}{{}^{\prime },\;\sigma }_{i})\in {\;L}_{S}$ or not, if so, B returns ${\;\sigma }_{i}$ to V, otherwise, selects a random $t_{i}{}^{\prime }\;\in {\;Z}_{q}^{*}$ and computes:
    $$\begin{array}{l} {A{}^{\prime }}_{i}{\;=\;t{}^{\prime }}_{i}\;P\;,\\ {r{}^{\prime }}_{i\;}{=\;F(\;A{}^{\prime }}_{i}\;)\;,\\ {y{}^{\prime }}_{i}{\;=\;r{}^{\prime }}_{i}{}^{-1}{\;(H(m}_{i}{)\;-\;H(m{}^{\prime }}_{i}{\;)\;+\;r}_{i}y_{i}\;)\;\mathrm{mod}\;q\;,\\ {Y{}^{\prime }}_{i}{\;=\;y{}^{\prime }}_{i}\;P\;,\\ k_{i}{\;=\;t}_{i}{{}^{\prime }-\;y}_{i}{\;f\;(h,\;r}_{i}{{}^{\prime },\;Y}_{i}{}^{\prime }\;)\;\mathrm{mod}\;q \end{array}$$
  Then, B generates individual signature ${\;(r}_{i}{{}^{\prime },\;k}_{i})$ and returns to V.
- *Forge*: After polynomial bounded queries, the attacker V outputs the aggregate signature $\sigma^{*}\;=\;(K^{*},\;C^{*})$ of the new message set ${\left\{m_{i}{}^{\prime *}\right\}}_{1}^{n}$ under the condition of the user’s long-term hash key set ${\left\{Y_{i}*\;\right\}}_{1}^{n}$ and the original message/auxiliary parameter set ${\left\{m_{i}{*,\;r}_{i}*\;\right\}}_{1}^{n}$, and satisfies the following two conditions at the same time:
  – $C\;=\;KP\;+\;\sum_{i=1}^{m}{\;f}_{i}Y_{i}$;
  – There is at least a message $m_{i}$ (m*) to which neither a key query nor an individual signature query is performed.

According to the Forking Lemma [31], the attacker V simply replaces the hash function f with $\tilde{f}$, a new valid forged signature $\tilde{\sigma }=({\tilde{K}}^{*},{\tilde{C}}^{*})$ is obtained. When j∈{1, 2, … , n}\{s}, there is s∈{1, 2, … , n} such that $f_{j}^{*}={\tilde{f}}_{j}^{*}$. When the following equations holds:

$$j\;=\;s,\;f(h^{*},{r_{j}}^{\prime }{}^{*},Y_{j}{}^{\prime }{}^{*})=f_{j}^{*}\neq {\tilde{f}}_{j}^{*}=\tilde{f}(h^{*},r_{j}{}^{\prime }{}^{*},Y_{j}{}^{\prime }{}^{*})$$

we can obtain the following equation set:

$$\begin{array}{l} C^{*}{\;=\;K}^{*}P\;+\;\sum_{i=1}^{m}{\;f}_{i}^{\;*}Y_{i}\\ {\tilde{C}}^{*}={\tilde{K}}^{*}P+\sum_{i=1}^{n}{\tilde{f}}_{i}^{*}Y_{i} \end{array}$$

At the same time, the following calculation is available:

$$\begin{array}{l} K^{*}-{\tilde{K}}^{*}+\sum_{i=1}^{n}(f_{i}^{*}y_{i}-{\tilde{f}}_{i}^{*}y_{i})=\sum_{i=1}^{n}\left(t_{i}{}^{*}-{\tilde{t}}_{i}{}^{*}\right)\\ K^{*}-{\tilde{K}}^{*}+(f_{s}^{*}-{\tilde{f}}_{s}^{*})y_{s}=\sum_{i=1}^{n}\left(t_{i}{}^{*}-{\tilde{t}}_{i}{}^{*}\right)\\ y_{s}={\left(f_{s}^{*}-{\tilde{f}}_{s}^{*}\right)}^{-1}(\sum_{i=1}^{n}\left(t_{i}{}^{*}-{\tilde{t}}_{i}{}^{*}\right)+{\tilde{K}}^{*}-K^{*}) \end{array}$$

The probability of successful breaking of ECDLP by B is converted into the following three events:

(1) $E_{1}$ represents that algorithm B does not terminate at the query stage;
(2) $E_{2}$ indicates that V successfully forged aggregate signature; and
(3) $E_{3}$ indicates the successful application of Forking Lemma.

The probability of solving the ECDLP by algorithm B is as follows:

$$\begin{array}{l} Pr\left(E_{1}\;\cap {\;E}_{2}\;\cap {\;E}_{3}\right){=\;Pr(E}_{1}{)\;Pr(E}_{2}{|\;E}_{1}{)Pr(E}_{3}{|\;E}_{1}\cap {\;E}_{2})\\ {Pr(E}_{1})\;\geq \;\frac{1}{q_{k}}\\ {Pr(E}_{2}{|\;E}_{1})\;\geq \;\varepsilon \end{array}$$

According to the Forking Lemma [31], we can obtain the following equation:

$$\begin{array}{c} {Pr(E}_{3}{|\;E}_{1}\cap {\;E}_{2})\;\geq \;\frac{1}{10^{6}\sqrt{{\;q}_{f}}}\\ {\varepsilon {}^{\prime }\;=\;Pr(E}_{1}\;\cap {\;E}_{2}\;\cap {\;E}_{3})\;\geq \;\frac{1}{10^{6}q_{k}\sqrt{{\;q}_{f}}}\varepsilon \end{array}$$

The running time of B is equal to the sum of V’s running time, B’s answer querying time and B’s time to successfully break the ECDLP instance with forged signatures. One key query, trapdoor hash query, and signature query respectively requires 1, 2, and 2 scalar multiplication on the group, so we can obtain:

$$t{}^{\prime }\;\leq {\;t\;+\;O(q}_{k}{\;+\;2q}_{T}{\;+\;2q}_{s}{)T}_{ME}.$$

In summary, the aggregate signature scheme proposed in this paper is ${(t}_{i}{{}^{\prime },\;\varepsilon {}^{\prime },\;q}_{H}\;{,\;q}_{K\;}{,\;q}_{T\;}{,\;q}_{S\;},\;n)$ existing unforgeable under adaptively chosen message attack. □

### 6.4. Security Comparisons

As shown in Table 3, the security of our MTH-AS scheme is compared with relevant aggregate signature schemes [11,24,35]. Since our proposed scheme selects the latest timestamp $t_{i}{}^{\prime }$, which is included in the messages ${{\{t}_{i}{{}^{\prime },\;r{}^{\prime }}_{i\;}{,\;A{}^{\prime }}_{i}{\;,Y{}^{\prime }}_{i\;}{,\;\sigma }_{i}\;\}}_{1}^{n}$. Replay attacks can be found by checking the freshness of the timestamp. Thus, our proposed scheme is resistant against replay attacks. The schemes proposed in literature [11,24,35] are proven to be secure based on the hardness of Co-CDHP, CDHP, CDHP, respectively. As mentioned above, the MTH-AS scheme proposed in this paper is proven to be secure against the existing unforgeability under adaptively chosen message attacks assuming ECDLP is hard. Our MTH-AS scheme could provide message authentication by checking whether the equations ${\;k}_{i}{P+\;f\;(h,\;r}_{i}{{}^{\prime },\;Y}_{i}{{}^{\prime }\;)Y}_{i}{\;=\;A}_{i}{}^{\prime }$, ${F(\;A{}^{\prime }}_{i}{\;)\;=\;r{}^{\prime }}_{i}$ hold. Due to the above security analysis, our MTH-AS scheme is suitable for secure communications in IoT applications with limited computing power, storage capacity, and bandwidth.

### 6.5. Performance Analysis

In this subsection, performance analysis is mainly carried out from two aspects: the performance comparison of related aggregate signature schemes and the performance comparison of aggregate signatures in IoTs. These schemes are measured by communication cost and computation cost, which are considered in terms of the length of the aggregate signature and the computational complexity of the aggregation verification algorithm respectively. 

In this paper, we adopt the performance evaluation method in [39]. The experiments are taken on an Intel I7 3.4 GHz, 4 GB machine with Windows 7 operating system. to obtain the security level of 80 bits, the bilinear pairing ${e\;:\;G}_{1}{\;\times \;G}_{1}\;\to {\;G}_{2}$ is conducted, where $G_{1}$ is an additive group generated by a point $\bar{P}$ with the order $q_{1}$ on the super singular elliptic curve $E_{1}{\;:\;y}^{2}{\;=\;x}^{3}{\;+\;x\;\mathrm{mod}\;p}_{1}$ (${\;p}_{1}$ and $q_{1}$ are 512-bit and 160-bit prime number, respectively) [36]. Accordingly the size of the elements in group $G_{1}$ (that is $L_{G_{1}}$) is 128 bytes (64 * 2 = 128). For ECC-based aggregate signature schemes, we use an additive group G generated by a point on a non-singular elliptic curve ${E\;:\;y}^{2}{\;=\;x}^{3}{\;+\;\mathrm{ax}\;+\;b\;\mathrm{mod}\;p}_{2}$ with the order $q_{2}$ ( $p_{2}$, $q_{2}$ are two 160-bit prime numbers,$\;a\;,\;b\;\in {\;Z}_{q}^{*}$ ). The size of the elements in group *G* (that is $L_{G}$ ) is 40 bytes (20 * 2 = 40). Let $L_{q}$ be the size of the elements in ${\;Z}_{q}^{*}$, *n* the number of signers. Table 4 lists the execution time of the encryption operations below [36,39].

(1) The comparison of aggregate signature schemes

**Figure 3:**
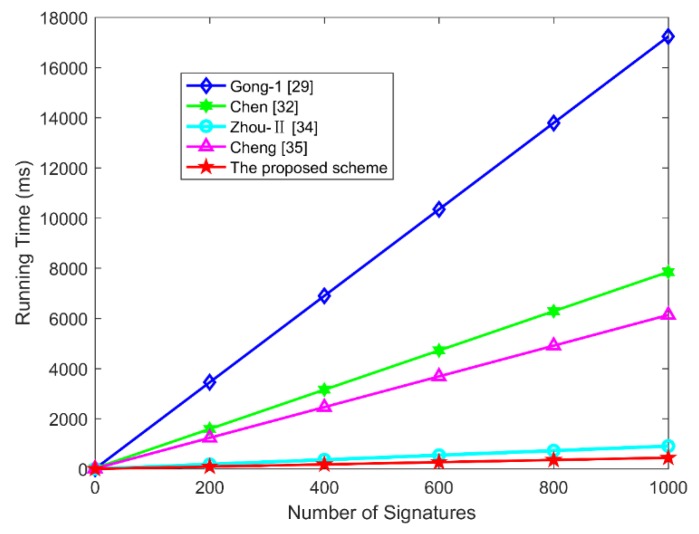
The comparison of aggregate verification time.

The scheme proposed in this paper is compared with the related aggregate signature schemes from four aspects: individual signature, aggregate verify, aggregate signature length and correlation between signature length and n. The specific performance comparison results of computation cost are presented in Table 5 and Figure 3.

As shown in Table 5, the individual signature time of the proposed scheme is 0.8843 ms, which is longer than that of CLAS-I [34]. However, the aggregate signature overhead of our scheme is better than that of CLAS-I [34]. The proposed scheme in this paper is based on ECC which is more efficient than bilinear pairings in computation cost [39]. Figure 3 shows that the aggregate verification time of our scheme is obviously better than that of the bilinear pairings based aggregate signature schemes [29,32,35], but slightly higher than ECC based schemes [34]. 

**Figure 4:**
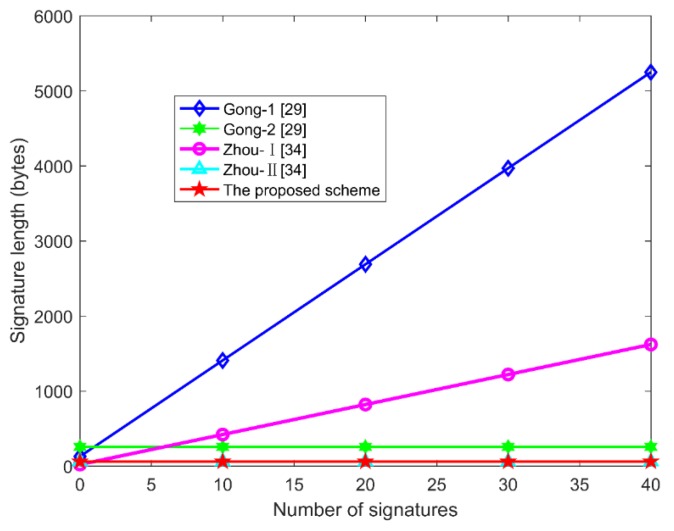
The comparison of signature length.

The comparisons of communication cost are presented in Table 6 and Figure 4. Since the size of $L_{G_{1}}$, $L_{G}$, $\;L_{q}$ are 128 bytes, 40 bytes, and 20 bytes, respectively, the lengths of Gong et al.’s *CAS*-1 and *CAS*-2 scheme [29] are 128 * n + 128 bytes and 2 * 128 = 256 bytes. The communication cost of Zhou et al.’s CLAS-I scheme [34] is 128 * n + 20 bytes. The proposed scheme in this paper has the same constant aggregate signature length as CLAS-II [34], which is 60 bytes, obviously superior to that of *CAS*-1 [29] and CLAS-I [34]. The proposed scheme in this paper has great advantages in communication efficiency.

Performance improvements are compared in Table 7. For example, compared to individual signatures of *CAS*-1 [29], the efficiency of performance improvement is approximately $\frac{7.8311-0.8843}{7.8311}\;\approx \;88.70\%$. Other performance improvements are calculated in the same way, assuming that the number of signatures is 50. As shown in Table 7, in terms of individual signature, the computation cost of our scheme is inferior to that of Zhou et al.’s CLAS-I scheme [34]. However, in terms of aggregate verify, the computation cost of our scheme is superior to all the other schemes [29,32,34,35]. Therefore, the proposed scheme in this paper is more efficient in aggregation verification.

(2) The comparison of aggregate signatures in IoTs

**Figure 5:**
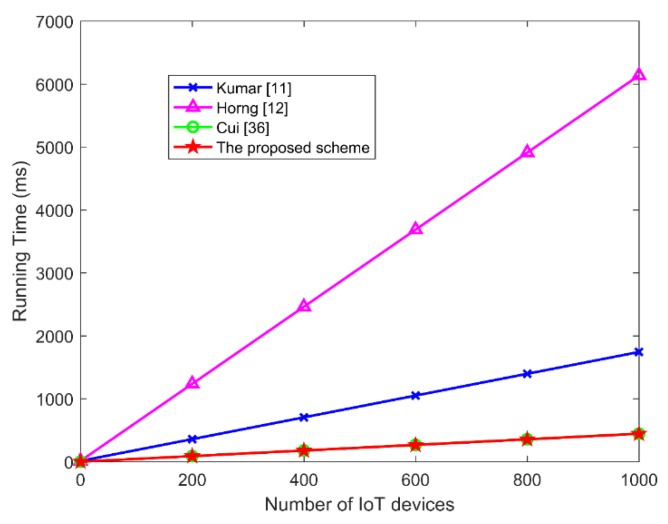
The comparison of aggregate verification cost in IoTs.

In IoT applications, it incurs lower computation cost and communication cost due to the limited battery capacity, computing power, bandwidth and storage capacity requirements of IoT devices. In this part, we compare the performance of the proposed scheme and other related IoT-based aggregate signature schemes. The cost of signature and verification is an important factor affecting the computing power of the IoT devices. Table 8 and Figure 5 show the comparison of computation cost in IoT-based aggregate signatures. From Figure 5, we can see that the aggregate verification delay of this proposed scheme is similar to that of Cui’s scheme [36], but is obviously superior to that of the other schemes [11,12], because bilinear pairings are not used in our scheme and Cui’s scheme [36]. 

**Figure 6:**
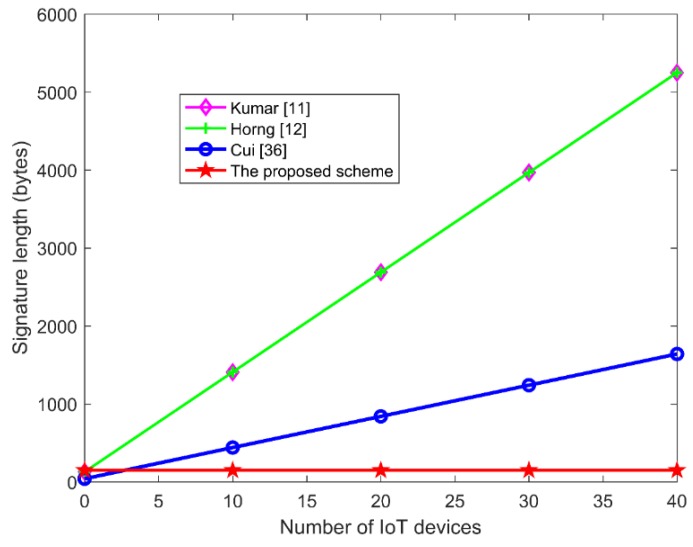
The comparison of signature length in IoTs.

The signature length affects the communication capability of IoT devices, which is an important factor in IoT applications. As shown in Table 9 and Figure 6, the signature length of our scheme is a constant (60 bytes), while the signature length of other schemes [11,12,36] are correlate with the IoT devices. In other words, as the number of the signed IoT devices increases, the signature length increases too. Therefore, the scheme proposed in this paper is more suitable for the application of IoTs because it has great advantages in saving bandwidth and storage capacity.

## 7. Conclusions

In this paper, an aggregate signature scheme is proposed based on ECDLP and MTH, which could be used for secure communication in many-to-one IoT applications. In the random oracle model, the proposed scheme is proven to be secure against the existing unforgeability under adaptively chosen message attacks. The proposed scheme has the characteristics of batch trapdoor collision calculation with multi-trapdoor hash functions and does not use bilinear pair operations. Therefore, it is more efficient than other schemes in terms of computation cost. On the other hand, the length of aggregate signature in this proposed scheme does not depend on the number of the signed IoT devices. Thus, the storage space and bandwidth are greatly saved. In summary, the scheme proposed in this paper is suitable for IoT applications with limited computing speed, storage capacity and bandwidth, such as wireless sensor networks, vehicular ad hoc networks, and healthcare sensor networks, etc.

## Figures and Tables

**Table 1:** The comparison of relevant aggregate signatures.

Scheme	Bilinear Pair	Constant Signature Length	Hardness Problem
Boneh [24]	Yes	No	Co-CDH
Gong-1 [29]	Yes	Yes	CDHP
Gong-2 [29]	Yes	No	CDHP
Chen [32]	Yes	Yes	CDHP
Zhou-I [34]	No	Yes	DLP
Zhou-II [34]	No	No	DLP
Cheng [35]	Yes	Yes	CDHP
Cui [36]	No	Yes	ECDLP,CDHP
Our proposed scheme	No	Yes	ECDLP

**Table 2:** Symbol description.

Symbol	Interpretation
|*x*|	Length of binary string *x*
$t\in_{R}V$	*t* is uniformly distributed in *V*
${\left\{\;x_{i}\;\right\}}_{m}^{n}$	$\left\{x_{m}{,\;x}_{m+1}{,\ldots ,\;x}_{n}\right\}$
${\left\{x\to \bar{x}\right\}}_{m}^{n}\backslash T$	$\left\{x_{m},\ldots ,x_{i-1},{\bar{x}}_{i},x_{i+1},\ldots ,x_{n}\right\}$, where ∀i∈T, T ⊂ {m,n}, $x_{i}={\bar{x}}_{i}$, other values of *x_i_* remain unchanged. For example, ${\left\{x_{i}\to {\bar{x}}_{i}\right\}}_{m}^{n}\backslash \{p,q\}$ represents $\left\{x_{m},\ldots ,x_{p-1},{\bar{x}}_{p},x_{p+1},\ldots ,x_{q-1},{\bar{x}}_{q},x_{q+1},\ldots ,x_{n}\right\}$.

**Table 3:** The security comparison of relevant aggregate signatures.

Scheme	Hardness Problem	Message Authentication	Resistance to Replay Attacks
Kumar [11]	CDHP	Yes	No
Boneh [24]	Co-CDHP	Yes	No
Cheng [35]	CDHP	Yes	No
Our proposed scheme	ECDLP	Yes	Yes

**Table 4:** Different encryption operation execution time.

Encryption Operation	Description	Time (ms)
$T_{B}$	The bilinear pair operation	4.2110
$T_{MB}$	The scalar multiplication in the bilinear pair	1.7090
$T_{AB}$	The bilinear pair-to-midpoint addition	0.0071
$T_{HB}$	The hash-to-point operation in bilinear pair	4.4060
$T_{ME}$	The scalar multiplication in elliptic curve	0.4420
$T_{AE}$	The point addition operation in elliptic curve	0.0018
$T_{H}$	The general hash operation	0.0001

**Table 5:** The comparison of computation cost.

Scheme	Individual Signature Time	Aggregate Verification Time
Gong-1 [29]	${2T}_{MB}{\;+\;T}_{AB\;}{+\;T}_{HB}\;\approx \;7.8311ms$	$\left(2n\;+\;1\right){\;T}_{B}{\;+\;2n\;T}_{HB}$ ≈ 17.234n + 4.211ms
Gong-2 [29]	${3T}_{MB}{\;+\;2T}_{AB\;}{+\;2T}_{HB}\;\approx \;13.9532ms$	$\left(n\;+\;2\right){\;T}_{B}{\;+\;nT}_{MB}{\;+\;n\;T}_{AB\;}{\;+\;2n\;T}_{HB}$ ≈ 14.7391n + 8.422ms
Chen [32]	${4T}_{MB}{\;+\;2T}_{AB\;}{+\;2T}_{HB}{+\;2T}_{H\;}\;\approx \;15.6624ms$	${\;4T}_{B\;}{+\;2nT}_{MB}\;+\;\left(n\;+\;2\right)T_{HB}{\;+\;2nT}_{H\;}$ ≈ 7.8242n + 25.656ms
Zhou-I [34]	$T_{ME}{\;+\;T}_{H\;}\;\approx \;0.4421ms$	$\left(2n\;+\;1\right)T_{ME}{\;+\;4nT}_{AE}{\;+\;2nT}_{H\;}$ ≈ 0.9166n + 0.422ms
Zhou-II [34]	$T_{ME}{\;+\;nT}_{AE}{\;+\;T}_{H\;}\;\approx \;0.0071n\;+\;1.7091ms$	$\left(2n\;+\;1\right)T_{ME}\;+\;\left(3n\;+\;1\right)T_{AE}{\;+\;2nT}_{H\;}$ ≈ 0.9085n + 0.4438ms
Cheng [35]	${4T}_{MB}{\;+\;2T}_{AB}{\;+\;T}_{HB\;}\approx \;11.2562ms$	${\;3T}_{B\;}{+\;nT}_{MB}{\;+\;nT}_{AB\;}{\;+\;nT}_{HB}{\;+\;nT}_{H\;}$ ≈ 6.1222n + 12.633ms
Our proposed scheme	${2T}_{ME}{\;+\;3T}_{H\;}\approx \;0.8843ms$	$\left(n\;+\;1\right)T_{ME}\;+\;\left(n\;+\;1\right)T_{AE}{\;+\;2T}_{H\;}$ ≈ 0.4438n + 0.444ms

**Table 6:** The comparison of communication cost.

Scheme	Aggregate Signature Length	Correlation between Signature Length and *n*
Gong-1 [29]	${(n\;+\;1)L}_{G_{1}}$	Yes
Gong-2 [29]	${2L}_{G_{1}}$	No
Chen [32]	${(n\;+\;1)L}_{G_{1}}$	Yes
Zhou-I [34]	${\mathrm{nL}}_{G}{\;+\;L}_{q}$	Yes
Zhou-II [34]	$L_{G}{\;+\;L}_{q}$	No
Cheng [35]	${(n\;+\;1)L}_{G_{1}}$	Yes
Our proposed scheme	$L_{G}{\;+\;L}_{q}$	No

**Table 7:** The comparison of computational costs with other schemes.

Scheme	Individual Signature Time (%) (n=50)	Aggregate Verification Time (%)(n=50)
Gong-1 [29]	88.70	97.39
Gong-2 [29]	93.66	96.96
Chen [32]	94.35	94.57
Zhou-I [34]	−100.00	51.06
Zhou-II [34]	57.16	50.65
Cheng [35]	92.14	92.90

**Table 8:** The comparison of computation cost in IoTs.

Scheme	Individual Signature Time	Aggregate Verification Time
Kumar [11]	${3T}_{MB}{\;+\;2T}_{AB\;}{+\;T}_{HB}{+\;T}_{H\;}\approx \;9.5437ms$	${3T}_{B}{\;+\;nT}_{MB}{\;+\;3nT}_{AB}$ ≈ 1.7303n + 12.633ms
Horng [12]	${2T}_{MB}{\;+\;T}_{AB\;}{+\;T}_{HB}\;\approx \;3.4252ms$	${3T}_{B}{\;+\;nT}_{MB}{\;+\;nT}_{AB\;}{\;+\;nT}_{HB}{\;+\;nT}_{H\;}$ ≈ 6.1222n + 12.633ms
Cui [36]	$T_{ME}{\;+\;T}_{AE\;}{+\;T}_{H}\;\approx \;0.4439ms$	$\left(n\;+\;2\right)T_{ME}{\;+\;2nT}_{AE}{\;+\;2nT}_{H\;}$ ≈ 0.4458n + 0.884 ms
Our proposed scheme	${2T}_{ME}{\;+\;3T}_{H}\;\approx \;0.8843ms$	$\left(n\;+\;1\right)T_{ME}\;+\;\left(n\;+\;1\right)T_{AE}{\;+\;2T}_{H\;}$ ≈ 0.4438n + 0.444 ms

**Table 9:** The comparison of communication cost in IoTs.

Scheme	Aggregate Signature Length	Correlation between Signature Length and *n*
Kumar [11]	${(n\;+\;1)L}_{G_{1}}$	Yes
Horng [12]	${(n\;+\;1)L}_{G_{1}}$	Yes
Cui [36]	${(n\;+\;1)L}_{G}$	Yes
Our proposed scheme	$L_{G}{\;+\;L}_{q}$	No
